# LATE EVALUATION OF PATIENTS OPERATED FOR GASTROESOPHAGEAL REFLUX DISEASE BY NISSEN FUNDOPLICATION

**DOI:** 10.1590/0102-6720201600030001

**Published:** 2016

**Authors:** Maxwel Capsy Boga RIBEIRO, Amanda Bueno de ARAÚJO, Juverson Alves TERRA-JÚNIOR, Eduardo CREMA, Nelson Adami ANDREOLLO

**Affiliations:** 1 Clinics Hospital and Department of Surgery, Faculty of Medicine, Federal University of Triângulo Mineiro, Uberaba, Brazil; 2 Program in Sciences of Surgery, State University of Campinas, Unicamp, Campinas, SP, Brazil

**Keywords:** Gastroesophageal reflux disease, Antireflux surgery, Fundoplication, Nissen

## Abstract

**Background:**

Surgical treatment of GERD by Nissen fundoplication is effective and safe, providing good results in the control of the disease. However, some authors have questioned the efficacy of this procedure and few studies on the long-term outcomes are available in the literature, especially in Brazil.

**Aim:**

To evaluate patients operated for gastro-esophageal reflux disease, for at least 10 years, by Nissen fundoplication.

**Methods:**

Thirty-two patients were interviewed and underwent upper digestive endoscopy, esophageal manometry, 24 h pH monitoring and barium esophagogram, before and after Nissen fundoplication.

**Results:**

Most patients were asymptomatic, satisfied with the result of surgery (87.5%) 10 years after operation, due to better symptom control compared with preoperative and, would do it again (84.38%). However, 62.5% were in use of some type of anti-reflux drugs. The manometry revealed lower esophageal sphincter with a mean pressure of 11.7 cm H_2_O and an average length of 2.85 cm. The average DeMeester index in pH monitoring was 11.47. The endoscopy revealed that most patients had a normal result (58.06%) or mild esophagitis (35.48%). Barium swallow revealed mild esophageal dilatation in 25,80% and hiatal hernia in 12.9% of cases.

**Conclusion:**

After at least a decade, most patients were satisfied with the operation, asymptomatic or had milder symptoms of GERD, being better and with easier control, compared to the preoperative period. Nevertheless, a considerable percentage still employed anti-reflux medications.

## RESUMO

**Racional:**

O tratamento cirúrgico para DRGE empregando a fundoplicatura à Nissen é eficaz e seguro, oferecendo bons resultados no controle da doença. Entretanto, alguns autores têm questionado quanto a sua eficácia, e poucos estudos com avaliação tardia destes doentes são encontrados na literatura, sobretudo no Brasil.

**Objetivo:**

Avaliar pacientes operados por doença do refluxo gastroesofágico há pelo menos 10 anos, pela técnica de Nissen.

**Métodos:**

Trinta e dois pacientes foram entrevistados e submetidos à endoscopia digestiva alta, manometria esofágica, pHmetria prolongada de 24h e esofagograma baritado, antes e após a fundoplicatura à Nissen.

**Resultados:**

A maioria estava assintomático 10 anos após a operação e satisfeitos com o resultado dela (87,5%) e a fariam novamente (84,38%), devido melhor controle sintomático com medicação, em comparação com o período pré-operatório. Entretanto, 62,5% empregavam algum tipo de medicação anti-refluxo. Quanto aos exames, a manometria revelou extensão média de 2,85 cm de esfíncter esofágico inferior, com pressão média de 11,7 cm de H_2_O. O índice médio de DeMeester na pHmetria foi de 11,47. Na endoscopia a maior parte dos pacientes apresentou exame normal (58,06%), ou esofagite leve (35,48%). O esofagograma revelou discreta dilatação esofágica em 25,80% e hérnia hiatal em 12,9% dos casos.

**Conclusão:**

Após no mínimo uma década, a maioria dos doentes estava satisfeita com a operação, assintomática ou apresentava sintomatologia mais branda da DRGE que era de melhor e mais fácil controle, comparado ao período pré-operatório. Porcentagem não desprezível ainda empregava medicações para refluxo.

## INTRODUCTION

Gastroesophageal reflux disease (GERD) is the most common digestive tract disorder in the western world^6^. It is defined when reflux of gastric content to the esophagus causes symptoms and complications^21^. It is estimated that 12% of the Brazilian population has GERD. Anamnesis is fundamental for the diagnosis of this condition and special attention should be paid not only to typical symptoms (heartburn and acid regurgitation), but also to atypical symptoms (oral, otorhinolaryngological, and pulmonary)^13^ ^,^ ^29^. 

The diagnosis of the disease can be confirmed by upper gastrointestinal (GI) endoscopy, contrast radiography of the esophagus, stomach and duodenum, esophageal manometry, and prolonged 24-hour esophageal pH monitoring^2^.

Surgical treatment of GERD by Nissen fundoplication is effective and safe, providing good results in the control of the disease^8^ ^,^ ^9^ ^,^ ^19^ ^,^ ^27^ ^,^ ^31^. Dysphagia, gas bloat syndrome and the inability to vomit are frequently reported by patients after surgery^22^ ^,^ ^26^. However, some authors have questioned the efficacy of this procedure^28^ and few studies on the long-term outcomes of these patients are available in the literature, especially in Brazil. In a recent meta-analysis, Garg and Gurusamy found only four studies with an appropriate design of analysis^11^. Spechler et al. reported good control of symptoms 10 years after surgery, although 62% of the patients still used some antireflux medication^30^ ^,^ ^32^. Lundell et al. found superior outcomes of surgical treatment compared to therapy with omeprazole^18^ ^,^ ^32^.

The objective of the present study was to evaluate patients operated for GERD by laparoscopic Nissen fundoplication after a minimum period of 10 years.

## METHODS

Thirty-two patients submitted to laparoscopic Nissen fundoplication at the University Hospital of the Federal University of Triângulo Mineiro, Uberaba, MG, Brazil, between 2000 and 2005 were evaluated. The study was approved by the Research Ethics Committee of the institution (Permit No. 2683/13). All patients had negative serology for Chagas' disease.

In addition to routine clinical evaluation, a specific questionnaire was applied to all patients to obtain information about typical and atypical symptoms of GERD. The patients were submitted to esophageal manometry, 24-hour esophageal pH monitoring, upper GI endoscopy, and barium esophagogram. 

Esophageal manometry was performed using an 8-channel catheter (Zynetics, Inc., Salt Lake City, UT, USA) connected to a pneumohydraulic infusion system (Arndorfer Medical Specialties, Greendale, WI, USA). The length of the lower esophageal sphincter (LES) and its resting pressure (cm H_2_O) were evaluated, as well as the amplitude of contractions of the esophageal body and the characteristics of its peristaltic waves. The resting pressure of the upper esophageal sphincter was also measured. 

In pH monitoring, the DeMeester score^7^ ^,^ ^16^ ^,^ ^23^ was used as a parameter, which takes into consideration six variables associated with GERD: number of acid reflux episodes, duration of these episodes, longest reflux duration, percentage of total reflux time, and percentage of reflux time in the upright and supine position. This test was performed without the use of any medication for GERD. Upper GI endoscopy was performed to evaluate the presence or absence of esophagitis and of complications of GERD. Esophagitis was classified as non-erosive or erosive using the Los Angeles criteria^12^. 

A barium esophagogram was used to evaluate the presence of hiatal hernia and tertiary waves, as well as esophageal diameter using the radiological classification proposed by Rezende^25^ as a reference.

## RESULTS

### Symptoms and satisfaction with surgery

Table 1 shows the percentage of each symptom determined with the GERD-specific questionnaire. When asked about satisfaction with the surgery, 87.5% of the patients were satisfied and 84.38% would have the operation again. Most of the patients with late symptoms confirmed improvement in the intensity of symptoms after surgery, reflecting the high proportion of patients who were satisfied with the long-term outcomes of surgery. None of the patients reported late dysphagia and late symptoms were described as sporadic, except for the difficulty burping and inability to vomit (Table 1).

**TABLE 1:** Incidence of late symptoms after Nissen fundoplication

Symptom	Incidence
Diurnal heartburn	40.63%
Nocturnal heartburn	18.75%
Acid regurgitation	31.25%
Burping difficulty	37.5%
Inability to vomit	34.38%
Atypical manifestations	12.5%

However, most of the patients of this study (62.5%) used, although irregularly, some medication (proton pump inhibitors, prokinetic agents, or both). Nevertheless, these patients reported better symptom control with the medication compared to the preoperative period. Table 2 shows the percentage of medications used in the late postoperative period. 

**TABLE 2:** Type and percentage of medications used in the late postoperative period after Nissen fundoplication

Medication	Incidence
No medication	37.5%
Proton pump inhibitor	50.01%
Prokinetic agent	9.38%
Proton pump inhibitor + prokinetic agent	3.13%

### Upper gastrointestinal endoscopy

Table 3 summarizes the findings of upper GI endoscopy classified according to the Los Angeles classification. As can be seen, 35.48% of the patients had mild non-erosive esophagitis and 3.23% developed late symptoms of moderate and severe esophagitis despite fundoplication.

**TABLE 3:** Incidence of esophagitis according to the Los Angeles classification in the late postoperative period after Nissen fundoplication

Grade of esophagitis	Incidence (%)
No esophagitis	58.06%
Mild esophagitis (non-erosive or Los Angeles A)	35.48%
Moderate esophagitis (Los Angeles B and C)	3.23%
Severe esophagitis (Los Angeles D and complications)	3.23%

### Esophageal manometry

The mean length of the LES was 2.85 cm and the mean resting pressure was 11.7 cm H_2_O. The mean amplitude of contraction waves in the esophageal body was 52.22 cm H_2_O and all patients exhibited peristaltic conduction waves during swallowing. Table 4 shows the findings of esophageal manometry in the late postoperative period after Nissen fundoplication.

**TABLE 4:** Manometry findings in the late postoperative period after Nissen fundoplication

Manometry findings	Incidence
Normal manometry	6.67%
Hypotony of LES	6.67%
Hypotony of UES	10%
hipocontractility of EB	13.33%
Combined hypotony of LES and hipocontractility of EB	23.33%
Combined hypotony of UES and hipocontractility of EB	10%
Combined hypotony of UES and LES	10%
Combined hypotony of UES, LES and hipocontractility of EB	20%

LES=lower esophageal sphincter; UES=upper esophageal sphincter; EB=esophageal body

### Prolonged 24-hour esophageal pH monitoring

The mean DeMeester score of the present sample was 11.47, a score below the reference value that characterizes pathological reflux (up to 14.92), ranging from 0.4 to 99.1. Only 20% of the patients had a high DeMeester score 10 years after surgery.

### Barium esophagogram

Table 5 shows the findings obtained according to the classification proposed by Rezende^25^. 

**TABLE 5:** Postoperative radiological findings of the esophagogram of patients submitted to Nissen fundoplication

Esophagogram	Incidence
Mild esophageal dilation	19.35%
Hiatal hernia alone	6.45%
Hiatal hernia associated with esophageal dilation	6.45%
Tertiary waves alone	3.23%

The mean esophageal diameter on the esophagogram was 2.85 cm. As can be seen in Table 4, only 6.67% of the patients studied had completely normal manometry findings, while the remaining patients exhibited manometric alterations in the esophageal body and sphincters. The results in Table 5 show that a significant percentage of the patients had postoperative radiological alterations. However, none reported any degree of dysphagia, a postoperative symptom that is the main cause of concern for surgeons after fundoplication.

## DISCUSSION

The short- and long-term outcomes of surgical treatment of GERD depend on different factors, including the indication for surgery because of clinical untreatability, adequate anamnesis, supplementary tests demonstrating the presence of gastroesophageal reflux, surgery observing the technical steps, and postoperative care and instructions^2^ ^,^ ^13^ ^,^ ^26^. 

The most common antireflux surgical technique is fundoplication as proposed by Rudolf Nissen in 1956^24^. The development and evolution of laparoscopic surgery has popularized surgical treatment of GERD^4^ ^,^ ^10^ ^,^ ^15^. Today, even robotic fundoplication can be performed safely with equivalent outcomes^20^.

Nissen fundoplication provides good short-term outcomes, although the risk of adverse events and complications is more prevalent than in clinical treatment^22^ ^,^ ^26^ ^,^ ^28^. Apparently, the long-term outcomes are also good, at least in terms of quality of life^1^ ^,^ ^5^ ^,^ ^14^, despite the need for some antireflux drug, a fact also observed in the present study. 

Here, clinically, most patients were very satisfied with the postoperative result. Even patients who required medication due to less intense and persistent gastroesophageal reflux reported easier control of reflux when compared to the preoperative period. Similar results have been reported by other authors with a good level of evidence^11^. 

At our Service, the Nissen procedure is performed as a short floppy fundoplication^8^ to avoid inconvenient symptoms of dysphagia and difficulty burping. The results of manometry analysis showed that the length and pressure of the LES were satisfactory even 10 years after the antireflux procedure. In addition, the amplitude of the contraction waves of the esophageal body exhibited a certain decrease, a finding that can be explained by the aging of this population. Although only a small proportion of the patients had completely normal manometry (6.67%) and most patients exhibited manometric abnormalities, these changes were not reflected in clinical worsening or the occurrence of dysphagia. 

In 24-hour pH monitoring, the DeMeester score was below the reference value in 80% of the patients 10 years after surgery and the mean score was within the normal range, suggesting good control of acid reflux in these patients many years after fundoplication. Another finding of this study was the absence of esophagitis in the majority of patients; however, if detected, milder forms were observed. Moreover, no complications related to GERD (peptid stenosis, Barrett's esophagus, or adenocarcinoma) were found. 

Morphologically, the barium esophagogram obtained revealed an intact esophagogastric transition zone in most cases 10 years after surgery, with topical fundoplication without hiatal hernias in 87.1% of the patients. Mild esophageal dilation considering the normal limit proposed by Rezende^25^ was observed in approximately one-quarter of the patients (25.08%). Pseudo-achalasia after fundoplication has been described in the literature^3^, a condition that could contribute to this dilation. All patients of this study had negative serology for Chagas' disease, although the disease is endemic in our region.

In the recent literature, several authors have demonstrated the advantages of Nissen fundoplication for the treatment of GERD. Amato et al. analyzed the long-term outcomes of fundoplication in 102 patients using the Short-Form 36 Health Survey and found that surgery offers improved quality of life, except for 5.8% of the patients who continued to have severe dysphagia^1^. Lundell et al., analyzing outcomes after 12 years of follow-up of 310 patients with GERD, concluded that antireflux surgery is superior to omeprazole in controlling manifestations of the disease, but some complaints continue even after fundoplication^18^. Rosemurgy et al.^27^ and Engstrom et al.^9^ who evaluated the long-term outcomes of laparoscopic fundoplication in 1,078 and 2,261 patients, respectively, reported that surgery promotes effective and durable treatment of GERD. 

On the other hand, Spechler et al., analyzing the outcomes of medical and surgical treatment for GERD after 10 years in randomized groups of 239 patients, found that 92% of the medical patients and 62% of the surgical patients still used antireflux medications regularly (p<.001). The conclusion of the study was that patients should be advised not to expect that surgery will mean that they will no longer need to take antireflux medications in the future^30^.

Sadowitz et al. evaluating the long-term outcomes of laparoscopic fundoplication in 100 patients with GERD, 84% rated the frequency of their symptoms as less than once a month, 88% were satisfied with the postoperative results, and 95% confirmed that they would have the operation again^28^.

Katada et al. analyzed the long-term effects of laparoscopic fundoplication on esophageal motility in 35 patients^17^. The authors observed that the LES pressures did not change significantly after surgery in the group with moderate esophagitis, but significantly increased in the group with severe esophagitis. The peristaltic wave amplitudes 18 and 13 cm above the LES did not change significantly after surgery in either group. The peristaltic contraction amplitudes 8 and 3 cm above the LES did not change significantly after surgery in the group with moderate esophagitis, but increased in the group with severe esophagitis. 

Finally, although late evaluation showed the need for antireflux medications in some cases, all patients had milder symptoms of GERD that were better and easier controlled at least a decade after surgery compared to the preoperative period. Furthermore, specialized workup showed a good length and pressure of the LES, a low incidence of pathological acid reflux, preserved fundoplication anatomy, a low incidence of severe esophagitis, and the absence of complications of GERD even several years after surgery, findings also observed by other authors. These results highlight the need for further well-designed studies for the long-term evaluation of Nissen fundoplication.

## CONCLUSION

After at least a decade, most patients were satisfied with the operation, asymptomatic or had milder symptoms of GERD, being better and with easier control, compared to the preoperative period. Nevertheless, a considerable percentage still employed anti-reflux medications. 
